# The Risk Factors for Complications Following Intestinal Stoma Reversal

**DOI:** 10.7759/cureus.97018

**Published:** 2025-11-16

**Authors:** Himanshu Goswami, Satish B Dharap, Dnyaneshwar K Mohare, Shantanu S Navgale, Piyush Kirange, Satyanand Singh

**Affiliations:** 1 General Surgery, Topiwala National Medical College & B. Y. L. Nair Charitable Hospital, Mumbai, IND

**Keywords:** anastomotic leak, complications, hartman’s procedure, intestinal stoma reversal, nutritional support, risk-factors, serum albumin

## Abstract

Background and objective

The restoration of bowel continuity after temporary intestinal stoma formation is a routine general surgical procedure. However, stoma reversal is associated with significant postoperative morbidity and mortality. Surgeons may face challenges such as dense adhesions, iatrogenic bowel injury, or even procedure abandonment, as well as postoperative complications such as surgical site infection (SSI), anastomotic leak, intra-abdominal sepsis, and death. This study aimed to identify common complications and their risk factors to facilitate strategies for optimizing surgical outcomes. Specifically, it sought to determine the incidence and pattern of early (within 30 days) postoperative complications, grade their severity using the Clavien-Dindo classification, and analyze the risk factors associated with complications following intestinal stoma reversal.

Methods

A prospective observational study was conducted in the Department of General Surgery at a tertiary care medical college hospital. All consenting adult patients undergoing reversal of a temporary intestinal stoma were included. Demographic, clinical, and operative variables - including age, sex, comorbidities, stoma type, indication, local pathology, interval between stoma creation and closure, preoperative chemo- or radiotherapy, hemoglobin, serum albumin, surgical technique, and use of postoperative nutritional support - were recorded. Outcome measures included intraoperative technical difficulty, iatrogenic bowel injury, postoperative complications, SSI, anastomotic leak, and mortality. Statistical analysis was performed using the Chi-square or Fisher’s exact test for categorical data and the *t*-test for continuous data. A *p*-value <0.05 was considered statistically significant.

Results

Seventy patients undergoing stoma reversal were included. Technical difficulty and iatrogenic bowel injury occurred in 23 (32.8%) patients and were significantly associated with colostomy reversal (*p*=0.002) and end stoma reversal (*p*=0.0059). Postoperative complications occurred in 32 patients (45.7%). The most common complication was SSI in 26 (37.1%), followed by anastomotic leak in six (8.6% ), intra-abdominal abscess in four (5.7%), abdominal wall dehiscence in four (5.7%), and enterocutaneous fistula in three (4.2%). There were four deaths (5.7%), all due to sepsis following anastomotic leak in patients with comorbidities. Preoperative serum albumin <3.5 g/dL was significantly associated with mortality (*p*=0.0007), while postoperative nutritional support significantly reduced complications (*p*=0.001).

Conclusions

Stoma reversal is linked to considerable morbidity and mortality; hence, the decision to create a diverting stoma should be made judiciously. Ileostomy reversal is technically easier and safer than colostomy or Hartmann’s reversal and may be preferred when diversion is indicated. Delayed reversal beyond three months, optimization of comorbidities, correction of hypoalbuminemia (>3.5 g/dL), and postoperative nutritional support are recommended to minimize complications and improve outcomes.

## Introduction

An intestinal stoma is the exteriorization of the bowel through the anterior abdominal wall. A temporary stoma is created to divert fecal flow, either to decompress obstructed intestines or to control sepsis in cases of intestinal perforation, most often in emergency settings. It may also be constructed prophylactically to protect a distal anastomosis when there is a high risk of leakage. Some surgeons routinely prefer a diverting stoma for all left-sided colorectal anastomoses. However, a stoma is associated with its own set of complications. Early postoperative issues include stomal necrosis, retraction, and detachment; intermediate problems include stomal granulation and ulceration; and late complications include skin excoriation, stomal stenosis, prolapse, and parastomal hernia [1]. In patients with a temporary stoma, reversal is generally planned once the anastomosis has healed and the risk of contamination has subsided, typically after two to three months.

Although often regarded as a minor operation, stoma reversal carries significant risks. Dissection and mobilization of the intestine may be difficult due to adhesions, and the procedure may need to be abandoned at times, as reported by Zehra et al. [2]. Dense adhesions can lead to inadvertent bowel injury, predisposing to intra-abdominal infection and sepsis. Postoperatively, anastomotic leakage may result in generalized peritonitis or intra-abdominal abscesses, which can progress to multiple organ dysfunction or even death. Enterocutaneous fistula formation, as demonstrated by Williams et al. [3], may further contribute to malnutrition, sepsis, and mortality. Contamination of the operative field may cause surgical site infection (SSI) and abdominal wound dehiscence. Systemic complications such as postoperative chest infections, deep vein thrombosis, and pulmonary embolism may also occur. Thus, a stoma reversal - often perceived as a straightforward operation - can be associated with serious morbidity requiring re-exploration, intensive care, or may even result in mortality.

Although stoma creation is frequently a life-saving procedure, its subsequent reversal entails considerable risk. Nelson et al. [4] reported that among patients with penetrating colonic injuries, mortality rates were similar between those undergoing primary repair and those receiving fecal diversion, suggesting that primary closure may be preferable in selected cases, given the complications associated with stoma reversal. In this context, and based on the hypothesis that "the incidence of major postoperative complications after the surgery of reversal of an intestinal stoma is underestimated”, the present study was undertaken to evaluate the intraoperative and postoperative complications associated with stoma reversal, to determine the incidence and pattern of overall and specific complications, and to identify related risk factors, with the ultimate aim of forming strategies for safer surgical practice.

## Materials and methods

This prospective observational study was conducted in the Department of General Surgery at a tertiary care medical college hospital in Mumbai, India, following approval from the Institutional Ethics Committee for Academic Research Projects (ECARP; Reference No. ECARP/2023/211). The study spanned from September 2024 to August 2025, encompassing the entire period from enrollment of the first participant to the final follow-up or completion of data collection for all patients. Each patient was followed up for 30 days after the surgical procedure.

All consecutive adult patients undergoing intestinal stoma reversal who provided informed consent and agreed to a 30-day postoperative follow-up were included. Patients younger than 18 years or unwilling to complete follow-up were excluded. Based on an expected anastomotic leak rate of 5% reported by Pokorny et al. (2005), the required sample size to estimate this proportion with 95% confidence and a precision of ±5% was calculated as 73 and rounded to 70 [5].

Independent variables included age, sex, comorbidities (diabetes mellitus, hypertension, chronic liver disease, chronic kidney disease, ischemic heart disease), type and site of stoma, interval between creation and closure (<3 months, three to six months, >6 months), indication for stoma formation, presence of local intestinal pathology (the presence of any intestinal lesion diagnosed on histopathology of the specimen retrieved at the time of stoma formation), history of preoperative chemotherapy or radiotherapy, preoperative hemoglobin (<10 g/dL or ≥10 g/dL), serum albumin (<3.5 g/dL or ≥3.5 g/dL), surgical technique (simple closure or resection with anastomosis), and use of postoperative parenteral nutrition.

The outcome measures were as follows: (1) intraoperative difficulty: defined as iatrogenic bowel injury or inability to identify the rectal stump; (2) postoperative complications: any deviation from the normal postoperative course, graded using the Clavien-Dindo classification (specific complications included SSI, abdominal wall dehiscence, intra-abdominal abscess, anastomotic leak, and enterocutaneous fistula; and (3) Mortality.

Statistical analysis was performed using IBM SPSS Statistics version 23 (IBM Corp., Armonk, NY). Associations between outcomes and independent variables were evaluated using the Chi-square or Fisher’s exact test for categorical variables, and the independent samples t-test or Mann-Whitney U test for continuous variables, as appropriate. A *p-*value <0.05 was considered statistically significant.

## Results

A total of 70 patients undergoing stoma reversal were included. Of these, 39 (56%) were males and 31 (44%) were females. The mean age of the cohort was 42 years (SD: 18, range: 13-82 years). Twenty (29%) patients had colostomy, and 50 (71%) had ileostomy. Thirty-two (46%) patients had a loop stoma, 31 (44%) had a double-barreled stoma, while seven (10%) had an end stoma (Hartmann type). Indications for stoma were exteriorization of perforated or gangrenous intestine in 35 (50%) patients, proximal diversion to protect a suture line in seven (10%), and diversion to relieve distal obstruction in 28 (40%). Among the 35 patients who received a stoma due to perforated or gangrenous bowel, 31 (44%) had localized disease that was excised during surgery, while four (6%) had traumatic perforations. Local bowel disease encompassed peritoneal or gastrointestinal tuberculosis, inflammatory bowel disease, typhoid or amoebic enteritis, and ischemic enteritis.

The mean time interval between stoma creation and reversal was 8.8 months (SD: 11.6 months), with a median interval of 7.5 months (range: 1-96 months, quartiles: 4, 11, and IQR: 7 months). As the time interval between stoma creation and reversal was not a parametric data, the time interval was divided into three categories (<3 months, three to six months, >6 months). Twenty-seven patients (38.5%) had at least one comorbidity, including diabetes, hypertension, asthma, ischemic heart disease, or end-stage renal disease. Ten patients (14.3%) had received chemotherapy and/or radiotherapy before stoma reversal. Serum hemoglobin levels ranged from 8.6 to 16.6 g/dL (mean: 11.53 g/dL; SD: 1.6 g/dL), with 11 patients (16%) having hemoglobin levels below 10 g/dL. Serum albumin levels ranged from 2.6 to 5.2 g/dL (mean: 3.83 g/dL; SD: 0.503 g/dL), and 13 patients (19%) had albumin levels below 3.5 g/dL.

Out of 70 patients who underwent stoma reversal, 23 (32.8%) suffered iatrogenic bowel injuries during surgery. This included two patients undergoing attempted Hartmann’s reversal in whom the procedure had to be abandoned because the distal rectal stump could not be identified. Consequently, stoma reversal was completed in 68 patients (97%). Of these 68 patients, 18 (26.4%) underwent simple closure, while 50 (73.5%) required resection and anastomosis. Postoperatively, 43 patients (61.4%) received parenteral nutritional support. Overall, 32 patients (45.7%) experienced complications following stoma reversal. Table 1 lists the various complications and their frequencies. Because some patients developed more than one complication, the total number of complications recorded was 43.

**Table 1:** Incidence of common postoperative complications (N=70)

Postoperative complications	N (%)
Surgical site infections	26 (37.1)
Abdominal wall dehiscence	4 (5.7)
Intra-abdominal abscess	4 (5.7)
Anastomotic leak	6 (8.6)
Enterocutaneous fistula	3 (4.2)

Table 2 presents the postoperative complications observed in 32 patients, classified according to the Clavien-Dindo grading system. Of these 32 patients, 18 (56.3%) required some form of intervention. Four patients (12.5%) who developed complications subsequently died.

**Table 2:** Postoperative complications as per the Clavien-Dindo grading system (n=32)

Grade of postop complication	N (%)
Grade I	3 (9.4)
Grade II	11 (34.4)
Grade III (a)	8 (25)
Grade III (b)	0 (0)
Grade IV (a)	4 (12.5)
Grade IV (b)	2 (6.3)
Grade V	4 (12.5)

Table 3 depicts the factors associated with iatrogenic bowel injury during reversal of stoma, which occurred in 23 out of 70 patients - a significantly high incidence in patients with colostomies and reversal of end stoma (Hartmann's procedure).

**Table 3:** Factors associated with iatrogenic bowel injury

Variables	Values	Iatrogenic bowel injury, n (%)	No iatrogenic bowel injury, n	Test used and value	P-value
Type of stoma (location)	Ileostomy	11(22)	39	Chi-square (9.35)	0.002
	Colostomy	12 (60)	8		
Type of stoma	Loop	7(21.9)	25	Fisher's exact	0.0059
	Double barrel	10 (32.3)	21		
	End	6 (85.7)	1		
Time interval between creation and closure	<3 months	2 (33.3)	4	Fisher's exact	0.75
	3-6 months	8 (27.6)	21		
	>6 months	13 (37.1)	22		
Local intestinal pathology	Present	9 (29.0)	22	Chi-square (0.347)	0.764
	Absent	14 (35.9)	25		
Preoperative chemo/radiotherapy	Present	5 (50)	5	Fisher's exact	0.18
	Absent	18 (30)	42		

Table 4 displays the factors associated with complications, noted in 32 of 70 patients (45.7%).

**Table 4:** Factors associated with complications SD: standard deviation

Variables	Values	Complication present, n (%)	No complication, n	Test used and test value	P-value
Age, mean, years		43 (SD: 3.58)	42.34 (SD: 18)	T-test (0.14)	0.883
Gender	Male	17 (43.6)	22	Chi-square (0.16)	0.68
	Female	15 (48.4)	16		
Type of stoma (location)	Ileostomy	21 (42)	29	Chi-square (0.97)	0.32
	Colostomy	11 (55)	9		
Type of stoma	Loop	10 (31.2)	22	Fisher's exact	0.0623
	Double barrel	17 (54.8)	14		
	End	5 (71.4)	2		
Comorbidities	Present	15 (55.6)	12	Chi-square (1.72)	0.18
	Absent	17 (39.5)	26		
Time Interval between creation and closure	<3 months	5 (83.3)	1	Fisher's exact	0.131
	3-6 months	11(37.9)	18		
	>6 months	16 (45.7)	19		
Indications for stoma	Exteriorization of perforated or gangrenous bowel	20 (57.1)	15	Fisher's exact	0.54
	Diversion to protect the distal anastomosis	3 (42.9)	4		
	Diversion due to distal obstruction	12 (42.9)	16		
Local intestinal pathology	Present	11 (35.5)	20	Chi-square (2.35)	0.12
	Absent	21 (53.8)	18		
Preoperative chemo/radiotherapy	Present	4 (40)	6	Fisher's exact	0.48
	Absent	28 (46.7)	32		
Preop hemoglobin	<10 g/dL	5 (45.5)	6	Fisher's exact	0.6
	>10 g/dL	27 (45.8)	32		
Preop serum albumin	<3.5 g/dL	9 (69.2)	4	Chi-square (3.56)	0.059
	>3.5 g/dL	23 (40.3)	34		
Technique	Simple closure	6 (33.3)	12	Chi-square (2.33)	0.62
	Resection anastomosis	22 (44)	28		
Postoperative nutritional support	Present	13 (30.2)	30	Chi-square (10.77)	0.001
	Absent	19 (70.4)	8		

Among 13 patients who had albumin <3.5 g/dL, nine (69.2%) developed postoperative complications; although this was not statistically significant (*p*=0.059), it showed a clinically relevant trend, as patients receiving postoperative nutritional support had a significantly lower incidence of postoperative complications. Surgical site infection (including abdominal wall dehiscence) was observed in 26 (37.14%) patients. Table 5 displays the factors associated with surgical site infections.

**Table 5:** Factors associated with surgical site infections SD: standard deviation

Variables	Values	Surgical site infection present, n (%)	No surgical site infection, n	Test used and test value	P-value
Age, mean, years		41.73 (SD: 18.4)	43 (SD: 18.18)	T-test (0.31)	0.75
Gender	Male	14 (35.9)	25	Chi-square (0.06)	0.8
	Female	12 (38.7)	19		
Type of stoma (location)	Ileostomy	17 (34)	33	Chi-square (0.74)	0.38
	Colostomy	9 (45)	11		
Type of stoma	Loop	7 (21.8)	25	Fisher's exact	0.04
	Double barrel	15 (48.4)	16		
	End	4 (57.1)	3		
Comorbidities	Present	11 (40.7)	16	Chi-square (0.24)	0.624
	Absent	15 (34.9)	28		
Time interval between creation and closure	<3 months	4 (66.7)	2	Fisher's exact	0.279
	3-6 months	9 (31.0)	20		
	>6 months	13 (37.1)	22		
Indications for stoma	Exteriorization of perforated or gangrenous bowel	9 (25.2)	26	Fisher's exact	0.14
	Diversion to protect the distal anastomosis	3 (42.9)	4		
	Diversion due to distal obstruction	14 (50)	14		
Local intestinal pathology	Present	8 (25.8)	23	Chi-square (3.06)	0.08
	Absent	18 (46.2)	21		
Preoperative chemo/radiotherapy	Present	3 (30)	7	Fisher's exact	0.44
	Absent	23 (38.3)	37		
Preop hemoglobin	<10 g/dL	4 (36.4)	7	Fisher's exact	0.6
	>10 g/dL	22 (37.3)	37		
Preop serum albumin	<3.5 g/dL	7 (53.8)	6	Fisher's exact	0.14
	>3.5 g/dL	19 (33.3)	38		
Technique	Simple closure	4 (22.2)	14	Chi-square (2.23)	0.13
	Resection anastomosis	21 (42)	29		
Postoperative nutritional support	Present	11 (25.6)	32	Chi-square (9.86)	0.001
	Absent	15 (65.2)	8		

End stoma was significantly associated with surgical site infections, while postoperative nutritional support was a significant protective factor. Table 6 shows the factors associated with anastomotic leak diagnosed in six (8.57%) patients.

**Table 6:** Factors associated with anastomotic leak (among patients who underwent reversal of stoma) SD: standard deviation

Variables	Values	Anastomotic leak detected, n (%)	No anastomotic leak, n	Test used and value	P-value
Age, mean, years		50 (SD: 17.96)	41.93 (SD: 18.72)	T-test (1.02)	0.34
Gender	Male	3 (7.9)	35	Fisher's exact	0.55
	Female	3 (10)	27		
Type of stoma (location)	Ileostomy	3 (6)	47	Fisher's exact	0.18
	Colostomy	3 (16.7)	15		
Type of stoma	Loop	3 (9.4)	29	Fisher's exact	0.5
	Double barrel	2 (6.5)	29		
	End	1 (20)	4		
Comorbidities	Present	5 (20)	20	Fisher's exact	0.02
	Absent	1 (2.3)	42		
Time Interval between creation and closure	<3 months	2 (33.3)	4	Fisher's exact	0.088
	3-6 months	1 (3.4)	28		
	>6 months	3 (9.1)	30		
Indications for stoma	Exteriorization of perforated or gangrenous bowel	3 (8.6)	32	Fisher's exact	1
	Diversion to protect the distal anastomosis	0	7		
	Diversion due to distal obstruction	3 (10.7)	25		
Local intestinal pathology	Present	3 (9.7)	28	Fisher's exact	0.5
	Absent	3 (8.1)	34		
Preoperative chemo/radiotherapy	Present	1 (12.5)	7	Fisher's exact	0.5
	Absent	5 (8.3)	55		
Preop hemoglobin	<10 g/dL	2 (18.2)	9	Fisher's exact	0.247
	>10 g/dL	4 (7.0)	53		
Preop serum albumin	<3.5 g/dL	4 (30.8)	9	Fisher's exact	0.01
	>3.5 g/dL	2 (3.6)	53		
Technique	Simple closure	2 (33.3)	4	Fisher's exact	0.32
	Resection anastomosis	15 (40.5)	22		
Postoperative nutritional support	Present	3 (7)	40	Fisher's exact	0.38
	Absent	3 (12)	22		

The presence of comorbidity and the value of serum albumin below 3.5 g/dL were significantly associated with anastomotic leak. Out of six patients who had anastomotic leaks, three developed enterocutaneous fistula with abdominal wall dehiscence. Of these three, one required ICU admission and was successfully discharged, whereas the other two did not survive. Two additional patients with anastomotic leaks developed deep intra-abdominal infections and underwent laparotomy with the creation of a double-barrel stoma. One of these patients died, while the other recovered and was discharged after a three-day ICU stay. The remaining patient with an anastomotic leak developed generalized peritonitis and succumbed to septicemic shock and multi-organ dysfunction on postoperative day four.

Out of four patients who had intra-abdominal abscesses, two cases were secondary to an anastomotic leak. One abscess resulted from an infected hematoma and was managed with percutaneous drainage, while another occurred due to a missed iatrogenic bowel perforation, requiring laparotomy and creation of a diversion stoma. Four patients (5.71%) died. Table 7 outlines the factors associated with mortality.

**Table 7:** Factors associated with mortality SD: standard deviation

Variables	Values	Mortality, n (%)	Survived, n	Test used and value	P-value
Age, mean, years		53 (SD: 16.6)	42 (SD: 18.18)	T-test (2.29)	0.06
Gender	Male	1 (2.6)	38	Fisher's exact	0.24
	Female	3 (9.7)	28		
Type of stoma (location)	Ileostomy	2 (4)	48	Fisher's exact	0.31
	Colostomy	2 (10)	18		
Type of stoma	Loop	1 (3.1)	31	Fisher's exact	0.3
	Double barrel	2 (6.5)	29		
	End	1 (14.2)	6		
Comorbidities	Present	4 (14.8)	23	Fisher's exact	0.01
	Absent	0	43		
Time interval between creation and closure	<3 months	2 (33.3)	4	Fisher's exact	0.03
	3-6 months	1 (3.4)	28		
	>6 months	1 (2.9)	34		
Indications for stoma	Exteriorization of perforated or gangrenous bowel	3 (8.6)	32	Fisher's exact	0.75
	Diversion to protect the distal anastomosis	0	7		
	Diversion due to distal obstruction	1 (3.6)	27		
Local intestinal pathology	Present	3 (9.7)	28	Fisher's exact	0.22
	Absent	1 (2.6)	38		
Preoperative chemo/radiotherapy	Present	0	10	Fisher's exact	0.5
	Absent	4 (6.7)	56		
Preop hemoglobin	<10 g/dL	2 (18.1)	9	Fisher's exact	0.11
	>10 g/dL	2 (3.4)	57		
Preop serum albumin	<3.5 g/dL	4 (30.7)	9	Fisher's exact	0.0007
	>3.5 g/dL	0	57		
Technique	Simple closure	1 (5.5)	17	Fisher's exact	0.7
	Resection and anastomosis	3 (6)	47		
Postoperative nutritional support	Present	1 (2.3)	42	Fisher's exact	0.15
	Absent	3 (11.1)	24		

The presence of comorbidity, early stoma reversal before three months of formation, and serum albumin below 3.5 g/dL were significantly associated with mortality. Table 8 summarizes risk factors associated with the outcome variables.

**Table 8:** Summary of factors associated with outcome measures

Outcome variables	Statistically significant risk factors
Iatrogenic injury	Colostomy, end stoma
Complication	Absence of postoperative nutritional support
Surgical site infection	End stoma, absence of postoperative nutritional support
Anastomotic leak	Presence of comorbidities, low serum albumin
Mortality	Presence of comorbidities, low serum albumin, and early stoma reversal

## Discussion

Complications following stoma reversal were observed in 32 (45.7%) patients, including four (5.7%) who subsequently died. Among survivors, reversal could not be completed in two patients due to technical difficulty, and in two others, a new stoma was required to manage postoperative complications. Thus, successful stoma reversal was achieved in 62 of 70 patients (88.6%). The overall complication rate was relatively high (45.7%). Pokorny et al., in a literature review cited in their study, reported morbidity rates ranging from 2.4% to 50% [5]. The higher rate observed in the present study may be attributed to thorough documentation of minor complications through prospective data collection. Additionally, stomas were created under emergency conditions in 63 patients (90%), which may have contributed to the increased complication rate.

Overall, complications were significantly associated with the presence of comorbidities, reversal performed within three months of stoma formation, hypoalbuminemia (<3.5 g/dL), and reversal of colostomy or Hartmann’s procedure as compared to ileostomy closure. The use of postoperative parenteral nutritional support was associated with fewer postoperative complications. The mortality rate in this study was 5.7% (4/70). All deaths resulted from sepsis secondary to anastomotic leak. Significant predictors of mortality included hypoalbuminemia, early reversal, and the presence of comorbidities. Pokorny et al. (2005) reported a 3% mortality and identified anastomotic leak (5%) as the major cause of death [5]. Similar findings have been described by Baastrup et al. (2019), who noted malnutrition, hypoalbuminemia, and early closure as key risk factors for mortality [6].

Significant predictors of anastomotic leak in this study included hypoalbuminemia and comorbidities. Parthasarathy et al. also identified serum albumin as an important predictor, given its role in wound healing, collagen synthesis, and immune competence [7]. Patients with anastomotic leaks presented with systemic features of sepsis (tachypnea, hypotension, altered mentation), peritonitis (abdominal tenderness, guarding, rigidity), or feculent/bilious drainage from the wound or drain site. Contrast-enhanced CT (CECT) of the abdomen was the imaging modality of choice to detect anastomotic leaks or loculated intra-abdominal collections. Of six patients with anastomotic leaks, one developed florid septicemia and generalized peritonitis and died on postoperative day five. Two patients presented with localized peritonitis due to intra-abdominal abscesses; both underwent re-exploration, and one subsequently died. The remaining three developed enterocutaneous fistulae, of whom only one survived. These three patterns of presentation (generalized peritonitis, localized abscess, and enterocutaneous fistula) have also been described by Mortensen et al. [8].

Four patients developed intra-abdominal abscesses. Three required re-exploration and stoma reformation, resulting in one death, while the fourth was successfully treated with percutaneous drainage. Aldardeer et al. (2021) also reported intra-abdominal abscess as a common complication, often managed by percutaneous aspiration [9]. Mortensen et al. (2004) highlighted anastomotic leak as a major cause of abscess formation, frequently necessitating laparotomy for peritonitis and sepsis [8].

Reversal of colostomy, particularly Hartmann’s procedure, was significantly associated with iatrogenic bowel injury, indicating technical difficulty. Iatrogenic injury, used as a surrogate marker for intraoperative difficulty, was significantly more frequent during colostomy reversal (OR: 5.32, 95% CI: 1.7-16.2, *p*=0.0034), especially Hartmann’s reversal (OR: 16.23, 95% CI: 1.8-144.9, *p*=0.0126). In two patients, reversal was abandoned due to dense adhesions and pelvic fixation. Zehra et al. (2022) similarly reported that 6.9% of Hartmann’s reversals were abandoned due to dense adhesions [2]. Tilney et al.(2007), in a meta-analysis, found a lower incidence of overall complications following ileostomy reversal compared to colostomy reversal [10]. Although the present study found an ileostomy reversal to be technically simpler (significantly lower risk of iatrogenic bowel injury), the overall complications were similar to those following colostomy reversal. The most common complication in the present series was SSI. Some previous studies have reported a higher SSI rate following colostomy reversal compared to ileostomy [10,11], but this was not observed in our cohort.

This study has several limitations. It was a single-center study with a relatively small sample size (n=70), a relatively short follow-up period of 30 days, and a probable selection bias due to lack of randomization. Stomas were created for varied indications, including trauma, sepsis, and malignancy. Procedures were performed by multiple surgeons with differing levels of experience, introducing variability in technique and intraoperative judgment. These factors may have contributed to heterogeneity in outcomes.

## Conclusions

Stoma reversal is associated with a considerable risk of complications, and so the decision to create a diverting stoma should be made carefully. Ileostomy closure is technically simpler and safer compared to colostomy or Hartmann’s reversal, making it a preferred option for fecal diversion when feasible. Delaying stoma reversal beyond three months allows for nutritional optimization, better control of comorbidities, and improvement of serum albumin levels above 3.5 g/dL, all of which contribute to reduced postoperative morbidity. Early postoperative parenteral nutritional support is recommended, as it may further lower complication rates and enhance surgical outcomes.

## References

[1] Townsend CM, Beauchamp RD, Evers BM, Mattox KL (2022). Sabiston Textbook of Surgery: The Biological Basis of Modern Surgical Practice. 21st Edition. Sabiston Textbook of Surgery: The Biological Basis of Modern Surgical Practice. 21st ed. Philadelphia: Elsevier.

[2] Zehra S, Abbas MK (2022). Hartmann's reversal: a single-centre experience. Cureus.

[3] Williams LJ, Zolfaghari S, Boushey RP (2010). Complications of enterocutaneous fistulas and their management. Clin Colon Rectal Surg.

[4] Nelson R, Singer M (2002). Primary repair for penetrating colon injuries. Cochrane Database Syst Rev.

[5] Pokorny H, Herkner H, Jakesz R, Herbst F (2005). Mortality and complications after stoma closure. Arch Surg.

[6] Baastrup NN, Hartwig MF, Krarup PM, Jorgensen LN, Jensen KK (2019). Anastomotic leakage after stoma reversal combined with incisional hernia repair. World J Surg.

[7] Parthasarathy M, Greensmith M, Bowers D, Groot-Wassink T (2017). Risk factors for anastomotic leakage after colorectal resection: a retrospective analysis of 17 518 patients. Colorectal Dis.

[8] Chambers WM, Mortensen NJ (2004). Postoperative leakage and abscess formation after colorectal surgery. Best Pract Res Clin Gastroenterol.

[9] Aldardeer AA, Alsuity A, Mahmoud AG (2021). Early same admission closure of temporary bowel stomas: pros and cones. Int Surg J.

[10] Tilney HS, Sains PS, Lovegrove RE, Reese GE, Heriot AG, Tekkis PP (2007). Comparison of outcomes following ileostomy versus colostomy for defunctioning colorectal anastomoses. World J Surg.

[11] Liang MK, Li LT, Avellaneda A, Moffett JM, Hicks SC, Awad SS (2013). Outcomes and predictors of incisional surgical site infection in stoma reversal. JAMA Surg.

